# Cancer Evolvability Determines Therapy Outcomes

**DOI:** 10.1111/eva.70320

**Published:** 2026-10-25

**Authors:** Ranjini Bhattacharya, Anuraag Bukkuri, Robert A. Gatenby, Joel S. Brown

**Affiliations:** ^1^ Department of Integrated Mathematical Oncology H. Lee Moffitt Cancer Center and Research Institute Tampa Florida USA; ^2^ Department of Cancer Biology University of South Florida Tampa Florida USA; ^3^ Department of Mathematics City St. George's, University of London London UK; ^4^ Department of Radiology H. Lee Moffitt Cancer Center and Research Institute Tampa Florida USA; ^5^ Cancer Biology and Evolution Program Moffitt Cancer Center and Research Institute Tampa Florida USA

**Keywords:** cancer evolution, chemotherapy, evolutionary game theory, evolutionary medicine, evolvability, targeted therapy

## Abstract

Cancer progression following treatment failure is an evolutionary process in which therapy acts as a selection pressure driving Darwinian selection on heritable variation to favor resistant clones. Heritable variation is a key determinant of how rapidly tumors can adapt to therapy. Heritable variation arises through various mechanisms such as genetic mutations, epigenetics, chromosomal rearrangements, and copy number variations, resulting in a lack of replicative fidelity. We consider evolvability as a property or trait of the cancer cells that influences the rate at which fitness‐relevant heritable variation is generated. Using this as our definition, we present an evolutionary game‐theoretic model to evaluate how evolvability shapes resistance dynamics under two treatment modalities: targeted therapy and chemotherapy. We first compare cancer populations with fixed evolvabilities: low or high. Targeted therapy imposes a steep selection gradient, enabling rapid resistance evolution, while chemotherapy imposes a flatter selection gradient but drives tumors toward more extreme resistance strategies. We show that targeted therapy works better in cancers with low evolvability, whereas chemotherapy better controls cancers with high evolvability. We then extend the model to incorporate “plastic evolvability,” in which cancer cells can adjust their evolvability in response to therapy‐induced stress. In this case, cells fine‐tune the trade‐off between acquiring higher resistance and limiting the costs of resistance and evolvability. Plastic evolvability sustains a higher tumor burden than fixed‐evolvability. To address the challenge of plastic evolvability, we develop and simulate an evolutionary double bind using sequential cycles of chemotherapy and targeted therapy. With an appropriate sequence and timing, the double bind can drive cancers with plastic evolvability to extinction. Our results highlight the importance of evolvability in shaping treatment response and underscore the need to incorporate evolutionary principles into therapy design.

## 1 Introduction

Cancer is a disease shaped by evolutionary forces acting within the body. Cancer cells are constantly exposed to selection pressures in the tumor microenvironment as they compete for nutrients and space while avoiding host response. Cancer treatment represents an additional evolutionary selection pressure (Casás‐Selves and DeGregori 2011; Greaves 2015). Natural selection favors cells that survive and proliferate under these microenvironmental conditions, leading to the emergence of fitter variants (Darwin 1868). In the face of therapy, most cancer cells sensitive to therapy die. However, some cancer cells possess or acquire traits that provide drug resistance (Vasan et al. 2019). This then leads to relapse in patients after months or years in remission (Wang et al. 2019). Evolution is, therefore, the main cause of treatment failure and subsequent disease progression.

Natural selection requires: heritable variation (like begets like but with heritable changes), a struggle for survival, and a link between the two (Brown 2016). Therapy creates a strong selection pressure. Variants capable of surviving in a drug‐exposed environment are selected by natural selection (Bhattacharya et al. 2025). Resistance can be either intrinsic or acquired (Wang et al. 2019; Sharma et al. 2017). Intrinsic resistance arises when resistant subclones exist at low frequencies within the tumor prior to drug administration and expand after therapy eliminates sensitive cells. For instance, the presence of HER2‐overexpressing cells before treatment is associated with poor response to cisplatin therapy (Huang et al. 2016). Acquired resistance, on the other hand, emerges during treatment as cancer cells evolve new resistance traits. A common mechanism involves the upregulation of drug efflux pumps through increased expression of ABC transporters following therapy exposure (Chen et al. 2021; Wander et al. 2020) conferring multidrug resistance to diverse therapies (Lei et al. 2023).

The rate at which a cancer cell population evolves depends on both the strength of natural selection and the capacity to generate heritable phenotypic variation upon which selection can act. We will refer to this capacity as evolvability, broadly defined as the ability of a biological system to generate heritable phenotypic variants that enable future adaptation (Brown 2016; Bhattacharya et al. 2025; Kirschner and Gerhart 1998; Wagner and Altenberg 1996; Fisher 1930). In quantitative genetics, evolvability is often quantified as heritability through additive genetic variance (Houle 1992; Bukkuri and Brown 2021), where genetic mutations provide the principal source of heritable variation. In cancer, however, heritable phenotypic variation can arise through both genetic and nongenetic mechanisms, including mutations, copy number alterations, chromosomal rearrangements, epigenetic inheritance, transcriptional programs, and stable cell‐state transitions (Duesberg et al. 2000; Tomlinson et al. 1996; Esteller et al. 2024; Fabbri et al. 2021; Song et al. 2021; Gupta et al. 2019; Subhadarshini et al. 2023; França et al. 2024). Here, we use the term evolvability to refer to the property or trait of cancer cells to generate heritable phenotypic variation that can be acted upon by natural selection.

The heritable fidelity of cell division determines the generation rate of heritable phenotypic variation. The degree to which like begets like is influenced by pathways regulating mutation rates (Tomlinson et al. 1996), epigenetic reprogramming (Esteller et al. 2024), mRNA translation (Song et al. 2021), and phenotypic plasticity (Gupta et al. 2019). These mechanisms collectively determine the rate at which a population generates fitness‐relevant phenotypic variants (see Figure 1). Natural selection then determines which variants persist and expand. In a stable environment, where populations reside at or near a local fitness optimum, high evolvability leads to the generation of variants that have reduced fitness, imposing a cost to high evolvability. Conversely, following a major environmental perturbation such as therapy, high evolvability generates phenotypes better adapted to the perturbed environment. At extremely high rates of variation generation, however, deleterious variants can accumulate faster than selection can remove them, potentially resulting in mutational meltdown (Gabriel and Bürger 1994; Lynch et al. 1993). Recently, Tyczynska Weh et al. modeled evolvability by explicitly including high and low mutation rates in an agent‐based model. They showed how high mutation rates favor evolutionary rescue while also preventing the population from evolving to its fitness optimum (Tyczynska Weh et al. 2025). Importantly, evolvability should not be confused with evolutionary rate. Evolvability describes the population's capacity to generate heritable phenotypic variation, whereas the rate of evolution depends on both heritable variation and the strength of selection acting on that variation. Therefore, populations with greater evolvability are not necessarily fast evolving; rather, they possess a greater capacity to respond adaptively when subjected to shifting selection pressures.

**FIGURE 1:**
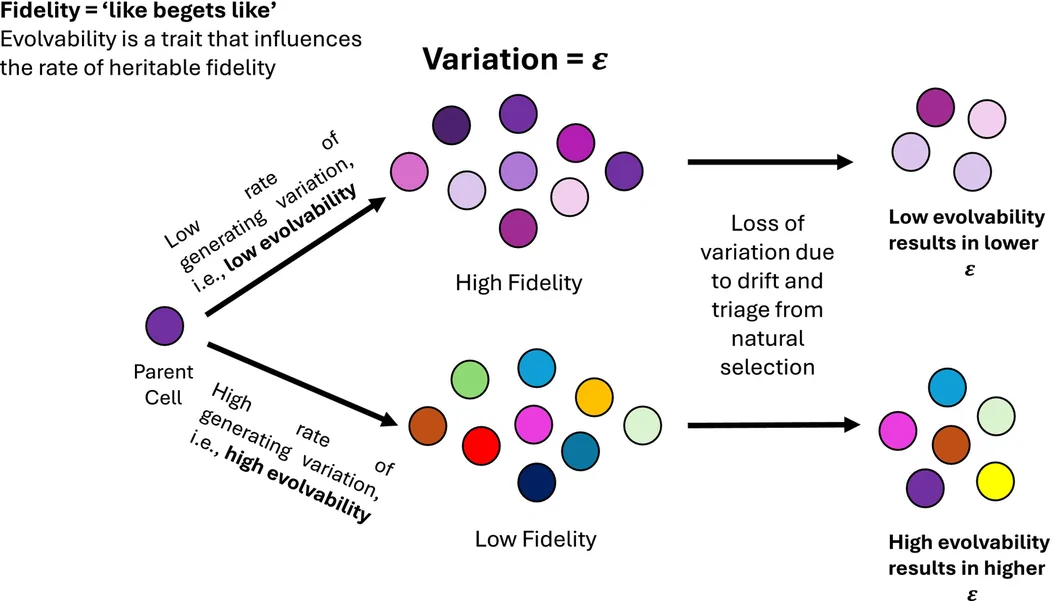
Conceptual relationship between evolvability, fidelity, and variation. Evolvability is a trait that determines the rate of lack of fidelity, or the degree to which “like begets like.” Low evolvability corresponds to high fidelity and leads to less variation, whereas high evolvability corresponds to low fidelity and generates more variation. In our model, this generated variation is represented by *ε*. Some of this variation can be lost through drift or selective triage.

Cancer drug therapies have diverse mechanisms for inducing cancer cell death, including cytotoxic treatments like chemotherapy (Anand et al. 2023) and targeted therapies (Min and Lee 2022; Joo et al. 2013). Chemotherapy typically involves a combination of cytotoxic drugs that interfere with biological processes such as DNA replication or cell division. These agents broadly affect all cells regardless of their phenotype and are associated with significant off‐target toxicity (Lowenthal and Eaton 1996). For instance, cisplatin forms DNA crosslinks that inhibit replication and transcription in both normal cells and cancer cells (Dasari and Tchounwou 2014). Because chemotherapy targets processes involved in cell cycling, it imposes a selective pressure that drives the emergence of higher and more heterogeneous resistance strategies. In contrast, targeted therapies are designed to inhibit specific molecular pathways that are implicated in cancer progression. These therapies are generally less toxic, with fewer off‐target effects, but are effective only on cells that rely on the targeted oncogenic driver (Shuel 2022). A well‐known example is tamoxifen, which binds competitively to the estrogen receptor and blocks estrogen‐mediated signaling in ER‐positive breast cancer (Shagufta and Ahmad 2018; Kim and Lukong 2025). While initially effective, targeted therapies are often vulnerable to resistance, as cancer cells need only activate an alternative signaling pathway to bypass the inhibited one. For example, resistance to tamoxifen can develop through upregulation of growth factor signaling pathways, such as HER2 or IGF1R, which can activate downstream proliferative signals independent of estrogen receptor activity (Chang 2012).

Here, we develop an evolutionary game‐theoretic model to investigate how evolvability influences a cancer population's response to chemotherapy and targeted therapy. A key distinction between these therapies is that chemotherapy is broadly effective across a wide range of cancer phenotypes but lacks specificity. In contrast, targeted therapy is highly specific, acting on a narrow subset of cancer cells.

We compare the therapeutic responses of three cancer population types: those with constant low evolvability, constant high evolvability, and plastic evolvability. Constitutive evolvability refers to cancer cells whose capacity to generate heritable phenotypic variation remains fixed regardless of environmental conditions. In contrast, we use the term plastic evolvability to describe populations whose evolvability can change in response to environmental conditions. The cells' capacity to generate heritable phenotypic variation increases in response to environmental stress (Fitzgerald et al. 2017; Pribis et al. 2022). Underlying cellular mechanisms that contribute to replicative heritable fidelity are activated during therapy but remain at a lower basal level in its absence. We use the term *plastic* to describe traits or processes that are conditionally expressed rather than fixed (Gupta et al. 2019; Pérez‐González et al. 2023). In cancer, such responses may include stress‐induced mutagenesis, epigenetic reprogramming, and phenotypic plasticity. These mechanisms operate within individual cells, influencing heritable variation at the population level.

We show that the three different populations respond differently to therapy. Based on the model's predictions, we offer therapeutic recommendations tailored to the cancer population's evolvability, with the goal of controlling the evolution of resistance and optimizing therapy outcomes.

## 2 Methods

### 2.1 Single‐Therapy Model

To investigate how evolvability influences the cancer population's response to therapy, we develop a *G*‐function model, a framework rooted in evolutionary game theory (EGT) (Vincent and Brown 2005; Smith 1982). The *G*‐function framework couples ecological dynamics (cell density) and evolutionary dynamics (trait distribution) (Bukkuri and Brown 2021). At the heart of this approach is a fitness‐generating function that defines how a population's fitness depends on both its traits and ecological context. In our model, we focus on how the fitness of a cancer cell population is shaped by drug efficacy and resistance traits (Bukkuri et al. 2023). We model resistance as a continuous trait. Equation (1) shows the *G*‐function, *G*(*v*, *x*), as a function of cell density (*x*) and the resistance strategy (*v*):

(1) $$\begin{array}{c} G\left(v,x\right)=\underset{\text{Logistic growth}}{\underbrace{r\left(1-\frac{x}{K\left(v\right)}\right)}}-\overset{\text{Drug}‐\text{induced death}}{\overbrace{s\exp\left(-\frac{v^{2}}{\sigma_{s}^{2}}\right)}}-\underset{\text{Cost of evolvability}}{\underbrace{\mathrm{d\varepsilon}\left(v\right)}} \end{array}$$

(2) $$K\left(v\right)=K_{m}\exp\left(-\frac{v^{2}}{\sigma_{k}^{2}}\right)$$

The first term in Equation (1) represents logistic growth, where *r* is the intrinsic per capita growth rate of the cancer cells and $K\left(v\right)$ is carrying capacity, modeled as a function of the resistance trait $v\in \left[0,\infty \right)$ (Equation 2). The maximum carrying capacity occurs when v=0 (no resistance) and decreases in a Gaussian manner as v deviates from 0. $\sigma_{k}$ represents this deviation. This formulation imposes an ecological cost of resistance, such that increased resistance reduces the carrying capacity of the population.

The second term in Equation (1) captures cell death from therapy. Drug efficacy is modeled as a Gaussian function (Bukkuri and Brown 2021), where s represents the maximum drug efficacy and $\sigma_{s}^{2}$ defines the breadth of drug sensitivity. Targeted therapy is modeled as highly specific (s=0.3) and effective over a narrow range of resistance values ($\sigma_{s}=\sqrt{2}$), whereas chemotherapy is less specific (s=0.17) but acts over a broader range of traits ($\sigma_{s}=\sqrt{6}$). The drug is most effective at v=0, and efficacy decreases as the resistance trait deviates from this value.

The third term in Equation (1) represents the cost of evolvability, modeled as the product of the population's evolvability ($\varepsilon \left(v\right)$) and a cost coefficient (*d*). This term captures the energetic and regulatory burden of generating heritable variation, such as the cost of increased mutation rates, epigenetic reprogramming, or investment in proteomic and transcriptomic plasticity (Zhang et al. 2018; Strobl et al. 2021). This cost may also be viewed as arising from the production of less‐fit phenotypes. Alternative ways of modeling this include using partial‐differential equations to describe the dynamics of the distribution of phenotypes in the population, thus building in the cost of evolvability (diffusion rate among phenotypes) explicitly (Chaplain et al. 2019).

### 2.2 Constant Evolvability

Next, we consider population and strategy dynamics. The population dynamics (Equation 3) is given by the product of the *G*‐function, $G\left(v,x\right)$, and the population density (x). The rate of evolution of the resistance strategy is constructed in accordance with Fisher's fundamental theorem of natural selection, according to which the rate of evolution is proportional to the trait's heritable variation, ε, and the selection pressure on the population, $\frac{\partial G}{\partial v}$ (Fisher 1930; Darwin 1859).

(3) $$\frac{\mathrm{dx}}{\mathrm{dt}}=\mathrm{xG}\left(v,x\right)$$

(4) $$\frac{\mathrm{dv}}{\mathrm{dt}}=\varepsilon \frac{\partial G}{\partial v}$$

The condition for eco‐evolutionary stability is then defined by: $\frac{\mathrm{dx}}{\mathrm{dt}}=\frac{\mathrm{dv}}{\mathrm{dt}}=0$ (Vincent and Brown 2005, 1988).

In our model, evolvability monotonically influences ε. We do not formally model the relationship between the rate at which heritable variation is generated and the amount of heritable variation, ε. Rather, we rely on the fact that heritable variation, ε, represents a balance between the rate of added variation and the loss of variability through drift and triage from natural selection. All else equal, higher, and lower evolvability will result in higher and lower ε, respectively (Figure 1). For the following simulations, ε=0.35 and ε=0.2 were used for high and low evolvability, respectively.

### 2.3 Plastic Evolvability

Here, we expand the model to consider plastic evolvability. One can imagine several ways to permit stress‐induced changes to a cell's heritable fidelity. We chose to let the rate of generating heritable variation increase with therapy‐induced stress. Therapy‐induced stress decreases as resistance levels increase; therefore, evolvability is modeled as a decreasing function of resistance, $\varepsilon \left(v\right)$:

(5) $$\varepsilon \left(v\right)=m+\mathrm{fs}\exp\left(-\frac{v^{2}}{\sigma_{s}^{2}}\right)$$

The constant m represents a basal level of evolvability in the absence of the stressor. Such conditions select for v=0, and heritable phenotypic variation arises from constitutive genetic and nongenetic processes (Payne and Wagner 2019; Nachman and Crowell 2000; Chalmers et al. 2017). The second term, $s\exp\left(-\frac{v^{2}}{\sigma_{s}^{2}}\right)$, represents the stress‐induced increase in evolvability during therapy, scaled by the factor f. We adjust the scaling factor to ensure that the maximum evolvability attained by a population with plastic evolvability does not exceed that of a population with constitutively high evolvability ($\varepsilon \;\left(\text{high}\right)=0.35$).

So, for choosing f, impose the following conditions:

(6) $$m+\mathrm{fs}\exp\left(-\frac{v^{2}}{\sigma_{s}^{2}}\right)\leq \varepsilon \;\left(\text{high}\right)$$

that is,

(7) m+fs≤0.35

We summarize the variables and parameters used in this model in Tables 1 and 2. The value of f will be different under chemotherapy and targeted therapy and can be found in Table 2.

**TABLE 1:** Summary of variables and symbols used in the single‐therapy model.

Symbol	Meaning
x	Cancer population size
v	Resistance strategy
$G\left(v,x\right)$	Fitness as a function of population size and resistance strategy
$K\left(v\right)$	Carrying capacity as a function of resistance strategy
$\varepsilon \left(v\right)$	Population evolvability for resistance strategy

**TABLE 2:** Summary of parameters used in the single‐therapy model.

Symbol	Meaning	Value
ε	Constant evolvability	$\left\{\begin{array}{c} 0.2,\text{if}\;\mathrm{low}\;\text{evolvability}\\ 0.35,\text{if high evolvability} \end{array}\right.$
f	Evolvability scaling factor	$\left\{\begin{array}{c} 1.91,\text{if chemotherapy}\\ 1.08,\text{if targeted therapy} \end{array}\right.$ (see Equation 7)
s	Therapy efficacy	$\left\{\begin{array}{c} 0.17,\text{if chemotherapy}\\ 0.3,\text{if targeted therapy} \end{array}\right.$
$\sigma_{s}$	Therapy efficacy breadth	$\left\{\begin{array}{c} \sqrt{6},\text{if chemotherapy}\\ \sqrt{2},\text{if targeted therapy} \end{array}\right.$
$K_{m}$	Maximum carrying capacity	100
r	Intrinsic growth rate	0.28
$\sigma_{k}$	Carrying capacity breadth	10
d	Cost of evolvability	0.127
m	Baseline evolvability	0.025

### 2.4 Dual‐Therapy Model

#### 2.4.1 Integrating Evolution of Multiple Resistance Strategies

In the initial model, we considered administration of only one drug. Next, we model the administration of two drugs. We modify the *G*‐function (Equation 1) to account for the two types of therapy, targeted therapy and chemotherapy.

(8) $$\begin{array}{c} G\left(x,v_{c},v_{t}\right)=\overset{\text{Logistic growth}}{\overbrace{r\left(1-\frac{x}{K\left(v_{c},v_{t}\right)}\right)}}-\overset{\text{Drug}‐\text{induced death}}{\overbrace{s_{c}\exp\left(-\frac{{v_{c}}^{2}}{\sigma_{s_{c}}^{2}}\right)-s_{t}\exp\left(-\frac{{v_{t}}^{2}}{\sigma_{s_{t}}^{2}}\right)}}-\underset{\text{Cost of evolvability}}{\underbrace{d\left(\varepsilon_{c}\left(v_{c}\right)+\varepsilon_{t}\left(v_{t}\right)\right)}} \end{array}$$

(9) $$K\left(v_{c},v_{t}\right)=K_{m}\exp\left(-\frac{v_{c}^{2}+v_{t}^{2}}{\sigma_{k}^{2}}\right)$$

In Equation (8), $v_{c}$ and $v_{t}$ represent distinct resistance strategies in response to chemotherapy and targeted therapy, respectively. The first term is the logistic growth term with a modified carrying capacity. The carrying capacity $K\left(v_{c},v_{t}\right))$ depends on both resistance strategies, $\left(\left(v_{c},v_{t}\right)\epsilon \;\left[0,\infty \right)\right)$ (Equation 9). $K\left(v_{c},v_{t}\right)$ is at its highest ($K_{m}$) when $v_{c}=v_{t}=0$. $K\left(v_{c},v_{t}\right)$ decreases as the two strategies diverge from 0 in a Gaussian manner. The second and third terms in Equation (8) are the chemotherapy and targeted therapy‐induced death terms, respectively. And the last term in Equation (8) is the cost of evolvability due to both drugs.

In the dual‐therapy model, we have two evolving traits. The rate at which meaningful heritable variation arises may be trait‐specific, as mutation rates or epigenetic changes can vary with traits. We build this into the model by allowing evolvability to differ between chemotherapy and targeted therapy‐induced selection.

When considering constant (constitutive) evolvability, we start by assuming that evolvability in both traits results in the same amount of trait‐specific heritable variation:

(10) $$\varepsilon_{c}=\varepsilon_{t}=\varepsilon$$

For plastic evolvability, we permit the degree of environmentally induced change in evolvability to be trait‐specific. Thus, the modulation of proliferative fidelity that generates heritable variation will differ for $v_{c}$ and $v_{t}$:

(11) $$\varepsilon_{c}\left(v_{c}\right)=m+f_{c}s_{c}\exp\left(-\frac{{v_{c}}^{2}}{\sigma_{s_{c}}^{2}}\right)$$

(12) $$\varepsilon_{t}\left(v_{t}\right)=m+f_{t}s_{t}\exp\left(-\frac{{v_{t}}^{2}}{\sigma_{s_{t}}^{2}}\right)$$

This distinction reflects the fact that different therapies activate distinct cellular stress‐response pathways and resistance mechanisms. For example, chemotherapy commonly induces DNA damage responses, stress‐induced mutagenesis, and multidrug resistance transporters, whereas targeted therapies often promote bypass signaling, pathway reactivation, or lineage plasticity (Wang et al. 2019; Lei et al. 2023; Anand et al. 2023; Joo et al. 2013; Shuel 2022; Bukowski et al. 2020). Consequently, the overall capacity of the population to generate heritable adaptive phenotypic variants may differ between therapeutic environments.

The resistance mechanisms that evolve in response to chemotherapy and targeted therapy could interact in three possible scenarios (Bukkuri and Brown 2021; Chou 2010): (1) The two drugs have independent modes of action, and resistance to one drug does not affect resistance to the other. In this case the two drugs are additive. (2) Resistance to one drug confers some resistance to the other drugs, that is, the two resistance strategies are positively correlated. This leads to cross‐resistance. Cross‐resistance reduces the efficacy of the two drugs (Bukowski et al. 2020; Loria et al. 2022). (3) Resistance to one drug makes cells more susceptible to the other drug. The two drugs act synergistically to enhance efficacy beyond additive effects (Chou 2010; Duarte and Vale 2022). In this case, the two resistance strategies have a negative covariance. Sequential application of such drugs leads to an evolutionary double bind (Gatenby et al. 2009). To model this, we introduce a covariance factor *δ* that captures the interdependence of the two resistance strategies (Bukkuri and Brown 2021). If *δ* = 0 the two resistance strategies evolve independently. If *δ* > 0, the two resistance strategies are positively correlated, and if *δ* < 0, the two strategies are negatively correlated (Equations 13 and 14).

(13) $$\frac{dv_{c}}{\mathrm{dt}}=\varepsilon_{c}\left(v_{c}\right)\frac{\partial G}{dv_{c}}+\delta \varepsilon_{t}\left(v_{t}\right)\frac{\partial G}{dv_{t}}$$

(14) $$\frac{dv_{t}}{\mathrm{dt}}=\varepsilon_{t}\left(v_{t}\right)\frac{\partial G}{dv_{t}}+\delta \varepsilon_{c}\left(v_{c}\right)\frac{\partial G}{dv_{c}}$$

An evolutionary double bind exploits collateral sensitivities between two drugs where resistance to one increases vulnerability to the other, creating a trap that can possibly drive the cancer population to extinction.

#### 2.4.2 Combination Therapy

For combination therapy, we use the same model outlined above, but in this case, both drugs are administered simultaneously.

### 2.5 Simulations

Therapy was administered at time step 600. Before administration of therapy, the resistance strategy does not evolve. The simulations were run until the population and strategy dynamics reached equilibrium. If the population size dropped below 1 at any point, we imposed an extinction threshold, setting the population to zero for all subsequent time steps. The variables and parameter values for the single‐therapy model are summarized in Tables 1 and 2, while those for the dual‐therapy model are summarized in Tables 3 and 4.

**TABLE 3:** Summary of variables introduced in the double‐bind model.

Symbol	Meaning
$v_{c}$	Resistance strategy for chemotherapy
$v_{t}$	Resistance strategy for targeted therapy
$G\left(x,v_{c},v_{t}\right)$	Fitness as a function of population size and two different resistance strategies
$K\left(v_{c},v_{t}\right)$	Carrying capacity as function of the two resistance strategies
$\varepsilon_{c}\left(v_{c}\right)$	Population evolvability under chemotherapy
$\varepsilon_{t}\left(v_{t}\right)$	Population evolvability under targeted therapy

**TABLE 4:** Summary of parameters used in the double‐bind model.

Symbol	Meaning	Value
$s_{c}$	Therapy efficacy of chemotherapy	0.17
$s_{t}$	Therapy efficacy of targeted therapy	0.30
$\sigma_{s_{t}}$	Targeted therapy efficacy breadth	$\sqrt{2}$
$\sigma_{s_{c}}$	Chemotherapy efficacy breadth	$\sqrt{6}$
$f_{c}$	Chemotherapy‐specific evolvability scaling factor	$\left\{\begin{array}{c} 1.91,\text{if full dose}\\ 3.82,\text{if half dose} \end{array}\right.$ (see Equation 7)
$f_{t}$	Targeted therapy‐specific evolvability scaling factor	$\left\{\begin{array}{c} 1.08,\text{if full dose}\\ 2.16,\text{if half dose} \end{array}\right.$ (see Equation 7)
δ	Covariance between two resistance strategies	−0.015

## 3 Results

### 3.1 Adaptive Landscapes

The adaptive landscape plots fitness, *G*, versus the strategy of a focal individual, *v*. The shape of the adaptive landscape is also influenced by population size *x*, rising and falling with decreases and increases in *x*, respectively. We examine how chemotherapy and targeted therapy shape their respective adaptive landscape; *G* versus $v_{c}$ and *G* versus $v_{t}$ (Figure 2). Evolutionary dynamics drive trait evolution up the landscape while ecological dynamics respond to whether *G* is positive (*x* increasing) or negative (*x* declining). In our example, at *x* = 20 a wide range of strategies result in positive population growth, including the fitness peak (Figure 2). As the population grows, an eco‐evolutionary equilibrium may be reached (*x*_eqb_, *v*_eqb_) where there is no further change in population size (*G* = 0) or strategy dynamics ($\frac{\partial G}{\partial v}=0$). At the eco‐evolutionary equilibrium, the fitness maximum coincides with *G* = 0 and all other strategies have negative fitness. In generating the adaptive landscape, we let targeted therapy have a higher therapeutic efficacy (*s*) but a narrower therapy efficacy breadth (*σ*_*s*_) than chemotherapy. This reflects the biological assumption that targeted therapies are highly specific and therefore effective against a relatively narrow range of cancer phenotypes, whereas chemotherapy acts across a broader range of cancer phenotypes. Consequently, targeted therapy (red curve) produces a steep reduction in fitness around the sensitive phenotype (*v* = 0), generating a strong local selection gradient favoring resistant variants. However, because therapeutic efficacy declines rapidly as resistance increases, populations possessing sufficient evolvability may generate resistant phenotypic variants capable of evolutionary rescue. In contrast, chemotherapy (blue curve) has a lower therapeutic efficacy but broader therapy efficacy across resistance strategies, producing a broader and shallower adaptive landscape. Selection for resistance is weaker. Achieving the same fitness benefit requires larger phenotypic shifts. This modeling assumption is motivated by the known differences in therapeutic specificity between targeted therapies and cytotoxic chemotherapy (Anand et al. 2023; Shuel 2022; Bukowski et al. 2020; Loria et al. 2022). The adaptive landscape under targeted therapy reaches its maximum at *v* = 2.82, whereas under chemotherapy the fitness optimum occurs at a higher resistance strategy (*v* = 3.87). Beyond these optima, fitness declines as the increasing cost of resistance outweighs the benefits of additional resistance. Consequently, chemotherapy selects for more extreme resistance strategies than targeted therapy, but these strategies are associated with larger fitness costs.

**FIGURE 2:**
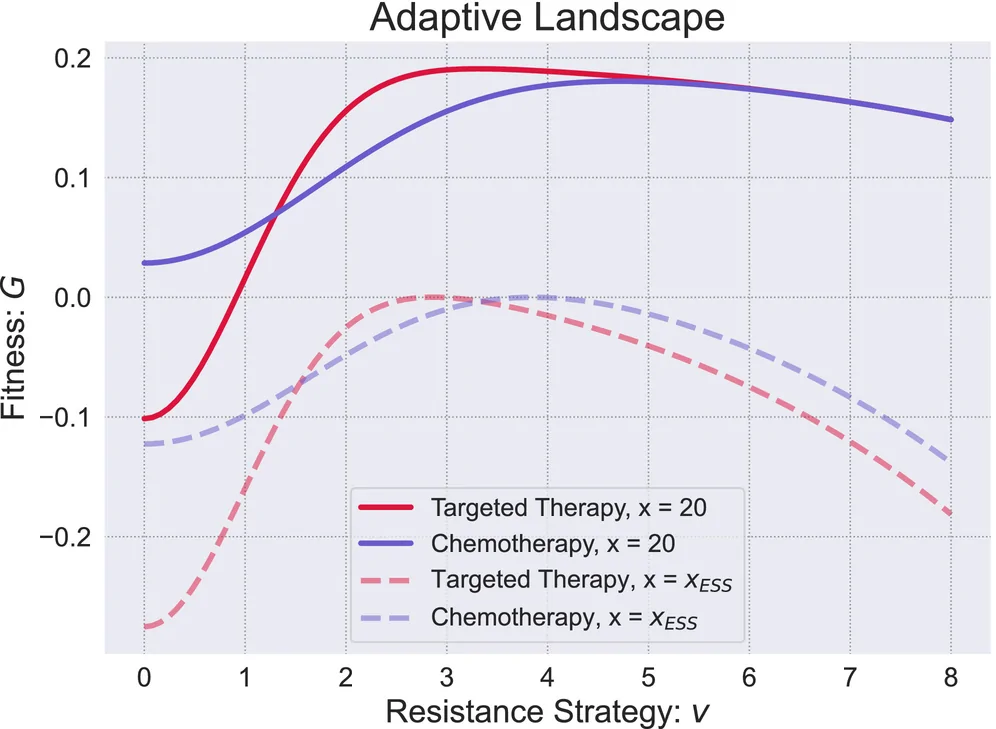
Adaptive landscape of cancer population under therapy: here, we show the adaptive landscape of the cancer population with fixed low evolvability, under chemotherapy (blue) and targeted therapy (red). Targeted therapy induces a steep selection gradient at strategies close to *v* = 0 and rapid evolution of resistance as the strategy values deviate from *v* = 0. Chemotherapy induces a gentler gradient but is effective on a broader range of resistance strategies. The adaptive landscape is drawn for a fixed population size. The solid lines correspond to, *x* = 20 and the dashed lines correspond to the adaptive landscape at equilibrium. For chemotherapy, *x*_eqb_ = 73.98 and for targeted therapy *x*_eqb_ = 82.10.

Therapeutic outcomes will depend on the interaction between the therapy‐induced selection gradient and the population's evolvability. We hypothesize that for cancer populations with low evolvability, targeted therapy may be more effective than chemotherapy due to its high efficacy at low resistance levels. Populations with high evolvability would speed the evolution of resistance to targeted therapy. Therefore, chemotherapy may be more effective in populations with high evolvability where fitness gains from evolving resistance are more modest. In the following sections, we will explore these therapy regimens.

### 3.2 Constant Evolvability

We consider targeted therapy and chemotherapy for populations with constant (constitutive) low and constant high evolvability (Figure 3). Prior to treatment, the population with constant low evolvability maintains a larger equilibrium tumor burden than the population with constant high evolvability because the intrinsic cost of evolvability reduces the effective carrying capacity (90.77 vs. 84.18; Figure 3a,b).

**FIGURE 3:**
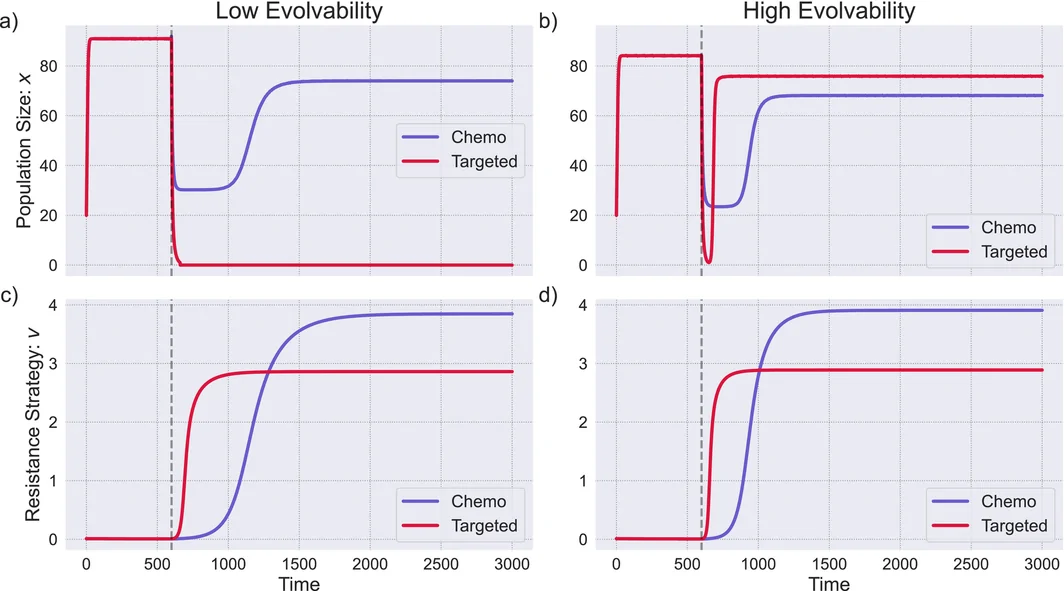
Dynamics of cancer populations with fixed evolvabilities: population dynamics (a, b) and resistance strategy dynamics (c, d) for the cancer population with low constant evolvability (a, c) and high constant evolvability (b, d). Therapy is administered at *t* = 600 (shown by the dashed vertical line). If the population size dropped below 1 at any point, we imposed an extinction threshold, setting the population to zero for all subsequent time steps. Without this extinction threshold, the population size at equilibrium for targeted therapy would be 82.10.

Following the initiation of targeted therapy, the population with constant low evolvability is unable to generate adaptive heritable phenotypic variation rapidly enough to escape the strong selection imposed by treatment. Tumor burden drops below the extinction threshold before evolutionary rescue can occur (Figure 3a,c). In contrast, the population with constant high evolvability generates resistant variants rapidly enough to undergo evolutionary rescue, converging on its eco‐evolutionary equilibrium (*x* = 75.79).

Under chemotherapy, both populations experience a shallow selection gradient and evolve toward the same resistance optimum (*v* ≈4). Because evolvability scales the rate of evolutionary change rather than altering the underlying adaptive landscape, both populations evolve toward the same equilibrium strategy values. Higher evolvability accelerates movement along the adaptive landscape without changing the equilibrium resistance strategy itself. A constant high evolvability increases the rate at which the population approaches this optimum, resulting in a shorter remission period than the population with constant low evolvability. However, because constant high evolvability also carries an intrinsic fitness cost through the evolvability penalty included in the model, the high‐evolvability population ultimately stabilizes at a lower equilibrium tumor size (68.11) than the population with constant low evolvability (73.98).

In summary, these results suggest that targeted therapy is most effective against populations with low evolvability, whereas chemotherapy suppresses populations with constitutively high evolvability by selecting for more costly resistance strategies (Figure 3).

### 3.3 Plastic Evolvability

We next compare the eco‐evolutionary trajectories of populations with constant low evolvability, constant high evolvability, and plastic evolvability (Figure 4). In our model, therapy‐induced stress transiently activates the cellular mechanisms that collectively modulate the evolvability of the population (Fitzgerald et al. 2017; Shi et al. 2023). While increased evolvability enhances the generation of resistant phenotypic variants, it also carries fitness costs associated with both maintaining elevated evolvability and evolving resistance. Consequently, populations with plastic evolvability balance the trade‐off between generating variation and minimizing the associated fitness costs (Enriquez‐Navas et al. 2015).

**FIGURE 4:**
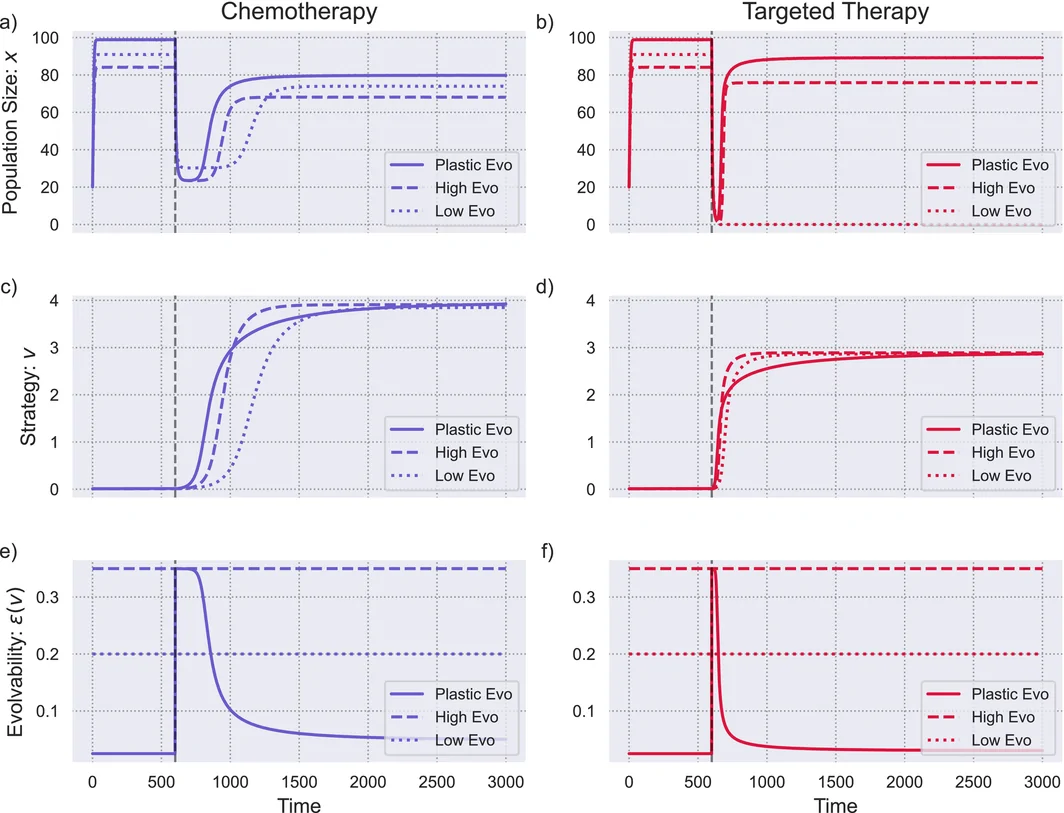
Comparison of dynamics with fixed and plastic evolvability: population dynamics (a, b), resistance strategy dynamics (c, d), and evolvability (e, f) dynamics of cancer populations with low, high, and plastic evolvabilities. Therapy is administered at *t* = 600 (indicated by the vertical dashed line).

Prior to treatment, the population with plastic evolvability maintains a lower basal evolvability. By remaining at this basal level in the absence of therapy, the population avoids continuously paying the intrinsic cost of elevated evolvability. Consequently, it achieves a higher effective carrying capacity (98.92) than populations with constitutively high evolvability, which incur this cost regardless of environmental conditions.

In our model, therapy‐induced stress accelerates the generation of heritable phenotypic variation enabling the rapid evolution of resistance. As resistance accumulates, therapy‐induced mortality decreases, reducing the selective advantage of maintaining elevated evolvability and evolvability gradually returns toward its basal level. This decline occurs more rapidly under targeted therapy than chemotherapy because, in our model, evolvability is coupled to therapy‐induced stress. Due to its higher therapeutic efficacy and narrower therapy efficacy breadth, targeted therapy‐induced stress decreases more rapidly once resistant variants emerge, resulting in a faster decline in evolvability than under chemotherapy.

Our simulations show that the plastic‐evolvability population attains a higher equilibrium population size than populations with fixed evolvability (79.77 in the case of chemotherapy and 89.21 in the case of targeted therapy). Plastic evolvability would increase the chances of therapy failure and relapse in patients.

### 3.4 Intermittent Therapy

To further explore the therapeutic implications of evolvability, we simulate intermittent therapy, where treatment is administered in periodic cycles rather than continuously. Intermittent therapy has been investigated clinically as a strategy to reduce toxicity, delay resistance, and prolong treatment efficacy (Tsai et al. 2017; Magnan et al. 2015). By periodically withdrawing therapy, this approach reduces the selective pressure that drives the emergence of resistant clones. In our model, the cyclic nature of intermittent therapy creates alternating periods of stress and recovery, and we examine how populations with fixed versus plastic evolvability adapt under this dynamic treatment regimen.

Figure 5 and Figure S1 show simulations for intermittent targeted therapy and chemotherapy, respectively, in the populations with constant low evolvability, constant high evolvability, and plastic evolvability. Therapy is administered at the 600th time‐step and removed and restarted every 50 time steps. During the “on therapy” periods, the population size decreases and resistance increases. During the “off therapy” or “drug holiday” periods, the cancer population bounces back, and the level of resistance decreases. This regular cycling between treatment and rest leads to fluctuations in both population size and resistance strategy dynamics. Intermittent therapy produces oscillations in both tumor burden and resistance strategy while preserving the qualitative differences observed under continuous therapy. For populations with constant evolvabilities, targeted therapy achieves near‐curative response. However, once therapy is revoked after 50 time steps, the population recovers and stabilizes into oscillations between a size ~81–85. A longer cycle duration (or continuous therapy as in Figure 3) would drive this population extinct. This highlights the importance of timing the drug holidays right.

**FIGURE 5:**
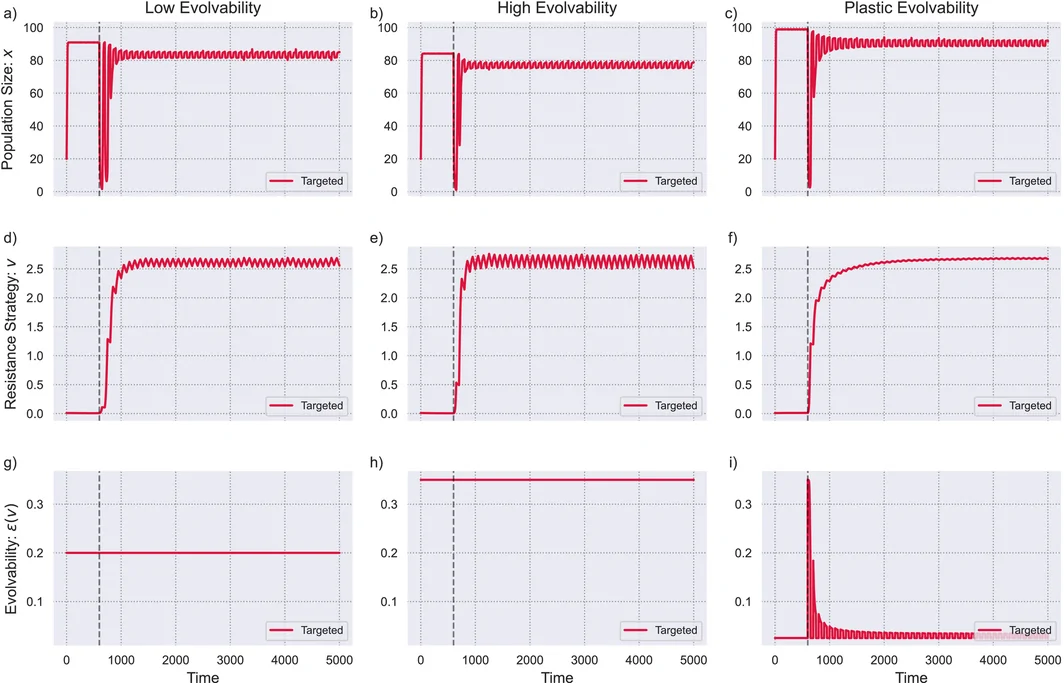
Comparison of dynamics under intermittent targeted therapy: population dynamics (a–c), resistance strategy dynamics (d–f), and evolvability (g–i) dynamics of cancer populations with low, high, and plastic evolvabilities under intermittent therapy. Therapy is administered at *t* = 600 (indicated by the vertical dashed line) and turned “on” or “off” every 50 time‐steps.

The cancer population with high constant evolvability oscillates between a population size of ~75–79 under targeted therapy. However, unlike the population with low constant evolvability, extending cycle duration does not drive this population to extinction (as seen in the continuous therapy case in Figure 3). Increasing therapy efficacy is the only way to eliminate it using a single drug.

Under intermittent chemotherapy (Figure S1), selection drives both populations with constant low and constant high evolvability toward higher resistance strategies than under targeted therapy, consistent with the adaptive landscape shown in Figure 2. As under continuous therapy, both populations oscillate around similar resistance strategies, with high evolvability primarily increasing the rate at which resistance responds to the alternating treatment cycles. Because the population with high evolvability also incurs the intrinsic fitness cost associated with maintaining elevated evolvability, it oscillates around a lower equilibrium population size (~66–76) than the population with constant low evolvability (~72–82). Consequently, chemotherapy achieves better long‐term tumor suppression in populations with high evolvability despite the similar equilibrium resistance strategies.

Consistent with the continuous therapy simulations (Figure 3), populations with plastic evolvability outperform both constant low‐ and constant high‐evolvability populations under intermittent therapy. Under targeted therapy, the population oscillates between equilibrium sizes of approximately 88–93, while under chemotherapy the oscillations occur over a slightly lower range of approximately 79–87. In both treatment regimens, populations with plastic evolvability maintain larger equilibrium tumor burdens than either constitutive strategy. Prior to therapy, these populations maintain a lower basal evolvability, thereby minimizing the intrinsic fitness cost associated with maintaining elevated evolvability. During treatment, therapy‐induced stress transiently activates the cellular mechanisms that regulate evolvability, increasing the population's capacity to generate adaptive heritable phenotypic variation. As therapy is withdrawn during the drug holiday, therapy‐induced stress declines and evolvability returns toward its basal level.

Intermittent therapy reveals the dynamic regulation of plastic evolvability in response to alternating periods of therapeutic stress and drug holidays. Unlike constitutive evolvability, cell populations with plastic evolvability can regulate the heritable variation they generate in response to alternating periods of therapy and drug holidays. Such intermittent treatment schedules are already used clinically to reduce toxicity, improve tolerability, and delay the emergence of resistance (Magnan et al. 2015). Despite repeated on–off treatment cycles, the population with plastic evolvability maintains a nearly constant resistance strategy with smaller oscillations than populations with fixed evolvabilities. Rather than continually adjusting its resistance strategy to each treatment cycle, the population maintains an intermediate resistance while transiently increasing evolvability only during therapy‐induced stress, thereby avoiding the continuous fitness costs of maintaining high evolvability. In contrast, populations with constitutive evolvability incur these costs regardless of environmental conditions.

### 3.5 Double Bind

Our next goal is to design a therapy regimen capable of driving the cell populations with constant high evolvability and plastic evolvability to extinction. To this end, we explore an evolutionary double bind—a strategy that traps the cancer population with no viable path to escape, much like a mouse caught between an owl in the open field and a snake in the bushes (Cunningham et al. 2011). This approach involves the sequential, cyclic administration of two drugs, such that resistance to one drug increases sensitivity to the other. Paramount to the design of an evolutionary double bind is the choice of drug order and cycle duration. The sequence in which drugs are administered may influence the rate at which resistance evolves and treatment efficacy. If therapies are switched too early, resistance may not have evolved sufficiently to create the double bind; if switched too late, resistant clones may escape. The interval between these extremes defines a cure window during which the second drug can eliminate the remaining vulnerable cells before evolutionary rescue occurs.

The potential for double binds has been explored by combining chemotherapy with immunotherapy, and radiotherapy with immunotherapy (Luddy et al. 2026; Basanta et al. 2012). In this section, we simulate an evolutionary double bind using chemotherapy and targeted therapy to investigate how the order of drug administration and cycle duration influence the evolutionary dynamics of the cancer population. We reason that administering chemotherapy first may slow down the evolution of resistance, as suggested by the shallower selection gradient observed in the adaptive landscape (Figure 2). If targeted therapy is administered after chemotherapy, it could eliminate cancer cells before they have time to adapt. We test this strategy in Figure 6 using a cycle duration of 50 time steps.

**FIGURE 6:**
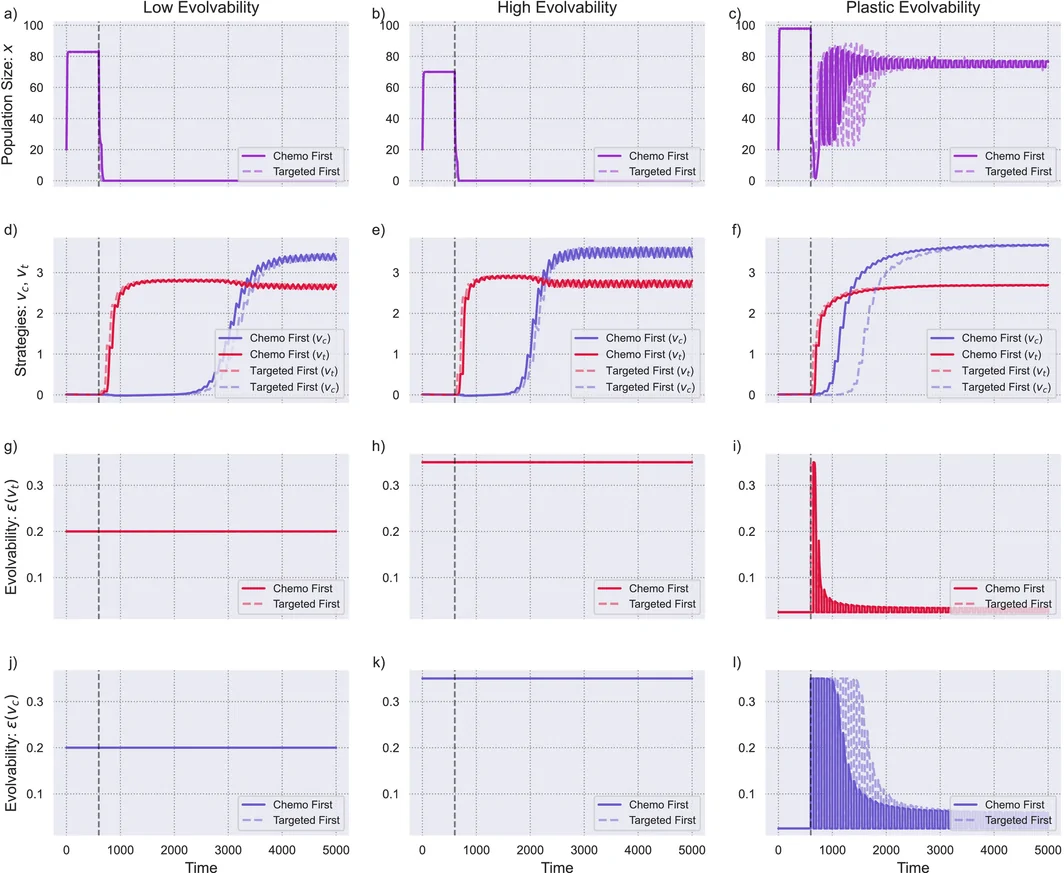
Comparison of dynamics under double‐bind therapy: population dynamics (a–c), resistance strategy dynamics (d–f), and evolvability (g–l) dynamics of cancer populations with low, high, and plastic evolvabilities under double‐bind therapy. Therapy is initiated at *t* = 600 (indicated by the vertical dashed line). Solid lines represent the regimen where chemotherapy is given first, followed by targeted therapy; dashed lines represent the reverse order. The cycle duration is short, that is, therapy is switched every 50 steps.

Prior to therapy, all three populations have lower effective carrying capacities in the double‐bind model because maintaining two resistance strategies and two evolvability traits imposes a greater cumulative fitness cost than in the single‐therapy model. The drop in effective carrying capacity is most pronounced in the cancer population with constant high evolvability (69.9 in the double bind vs. 84.18 in the continuous case), followed by the population with low constant evolvability (82.8 vs. 90.77), and finally the population with plastic evolvability (97.8 vs. 98.9). Interestingly, even before therapy begins, the population with plastic evolvability does better than the populations with constant evolvabilities. This is because it maintains evolvability at a baseline level, avoiding the higher costs incurred by constantly sustaining high evolvability.

Using a double bind with a cycle duration of 50 (short cycle), both populations with constant evolvabilities are driven to extinction. Chemotherapy slows down the evolution of resistance while reducing the population size. Subsequent administration of targeted therapy drives the population below the extinction threshold. Although the resistance strategies in Figure 6d,e converge to values similar to those under continuous or intermittent single‐drug therapy ($v_{c}\approx v_{t}\approx 3$), they have different consequences in the double‐bind model. Unlike the single‐drug model, the double‐bind model evolves two coupled resistance strategies ($v_{c}$ and $v_{t}$), and fitness depends on both resistance strategies. The same value of an individual resistance strategy does not imply the same fitness or growth rate. Because resistance to one therapy is coupled to increased sensitivity to the other, adaptation to the first drug creates a vulnerability that is exploited when treatment switches, ultimately driving the population to extinction.

This regimen, however, fails in populations with plastic evolvability. The two evolvability traits fluctuate with the treatment schedule: during chemotherapy, $\varepsilon_{c}\left(v_{c}\right)$ increases while $\varepsilon_{t}\left(v_{t}\right)$ remains near its basal level, and the reverse occurs during targeted therapy. With 50‐time‐step cycles, neither resistance strategy has sufficient time to evolve, preventing the cross‐sensitivity required for the double bind to be effective.

To allow this vulnerability to develop, we increased the cycle duration to 200 (Figure 7). The longer treatment period enables resistance to the first drug to evolve, establishing the negative covariance that the second drug exploits. Under this regimen, the plastic evolvability population is driven to extinction during the second cycle when targeted therapy follows chemotherapy. Reversing the drug order permits evolutionary rescue.

**FIGURE 7:**
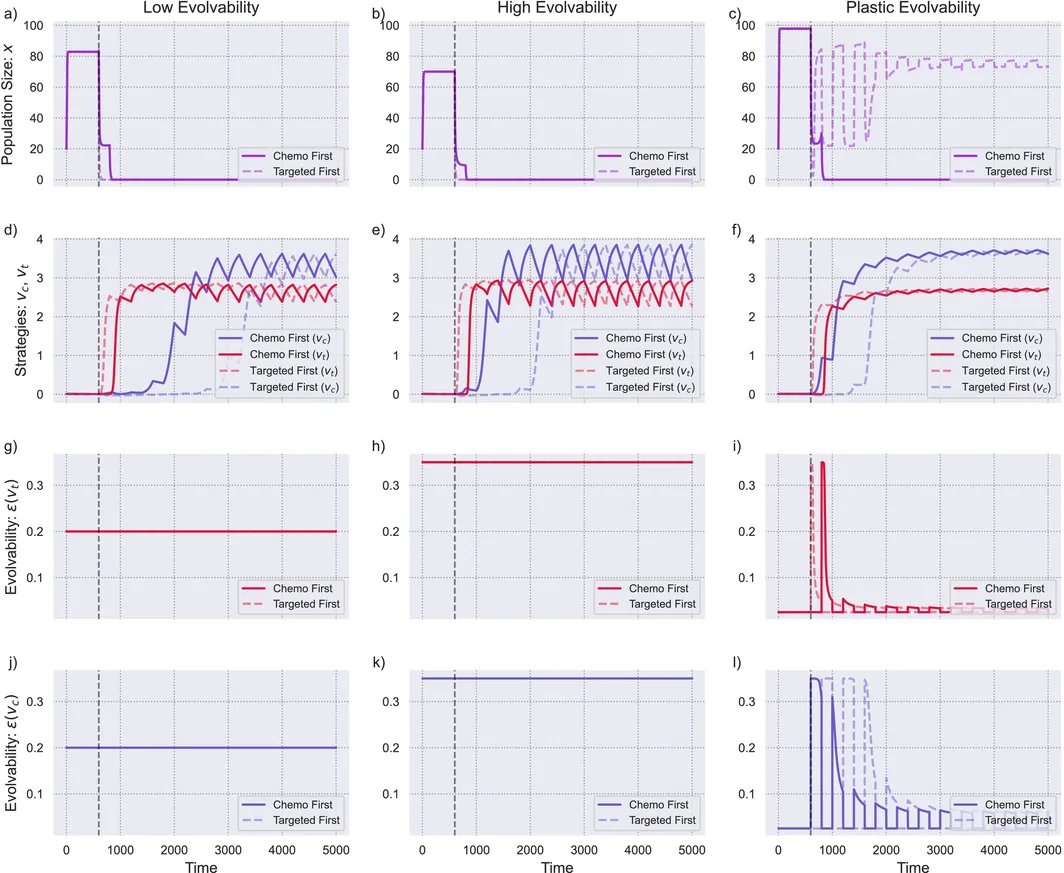
Comparison of dynamics under double‐bind therapy: population dynamics (a–c), resistance strategy dynamics (d–f), and evolvability (g–l) dynamics of cancer populations with low, high, and plastic evolvabilities under double‐bind therapy. Therapy is initiated at *t* = 600. Solid lines represent the regimen where chemotherapy is given first, followed by targeted therapy; dashed lines represent the reverse order. The cycle duration is long, that is, therapy is switched every 200 steps.

To facilitate comparison, we present results for combination therapy, where chemotherapy and targeted therapy are administered simultaneously, in Figure S2. Continuous administration of a drug cocktail without sequencing or switching is commonly used in clinical settings. At the full drug dose, all three populations go extinct. However, drug doses are often reduced clinically to limit toxicity. To reflect this, we also simulated treatment dynamics at half the full dose. Under these conditions, the population with constant high evolvability is still eliminated, whereas the populations with low and plastic evolvability escape cure. Although full‐dose combination therapy is the most effective regimen in our model, sequential double‐bind therapy achieves superior outcomes compared to half‐dose combination therapy while avoiding the toxicity associated with administering both drugs at full dose simultaneously.

We present a second example of double‐bind therapy in the Figure S1. Here, the cancer population has a higher intrinsic growth rate and a lower cost of evolvability, with fixed evolvability values of *ε* = 0.15 (low) and *ε* = 0.4 (high) (Table S1). With a 50‐time‐step cycle, the populations with low and plastic evolvability escape the double bind through evolutionary rescue (Figure S3). Increasing the cycle duration to 300 eliminates the populations with plastic evolvability but not the population with low evolvability (Figure S4), demonstrating that low evolvability can confer a survival advantage when the cost of evolvability is reduced. The plastic population transiently elevates evolvability in response to therapy, incurring a fitness cost that increases its susceptibility to the double bind.

The population with low evolvability can be eliminated by using combination therapy at the full drug efficacy (Figure S5). Extending the double‐bind cycle to 500 with chemotherapy administered first, followed by targeted therapy, drives all three populations to extinction (Figure S6).

Overall, cancer evolvability plays a critical role in shaping the response to therapy, and treatment strategies should be designed accordingly. By understanding the evolutionary dynamics of resistance and the influence of evolvability, we can make informed decisions about whether to combine therapies or sequence them. When sequencing drugs in a double bind, allowing the right amount of resistance to evolve is essential for setting the trap. This process is governed by the drug order, timing of the switch, and cycle duration, all of which must be carefully optimized to maximize treatment efficacy.

## 4 Discussion

In this study, we explored how cancer evolvability shapes therapeutic outcomes using an evolutionary game theory framework. Evolvability is a trait that influences the rate at which a population can generate heritable phenotypic variation upon which natural selection can act. Classical studies have shown that the capacity to generate adaptive variation depends on properties such as robustness, modularity, redundancy, developmental constraints, and genotype–phenotype mapping, which together permit the accumulation of heritable variation without compromising viability (Kirschner and Gerhart 1998; Wagner and Altenberg 1996; Houle 1992; Hansen et al. 2023; Ferrare and Good 2024; Bourrat et al. 2024). Analogous properties in cancer include redundant signaling pathways, chromosomal instability, stress‐response networks, epigenetic regulation, and plastic cell‐state programs (Esteller et al. 2024; Gupta et al. 2019; Bakhoum and Cantley 2018). Our framework abstracts their collective effects into a single evolvability trait that regulates the generation of heritable variation during therapy.

We specifically examined how resistance evolves under chemotherapy and targeted therapy. Targeted therapies act on specific oncogenic pathways and impose steep selection gradients that favor the rapid evolution of resistance (Shuel 2022). In contrast, chemotherapy targets broad cellular processes, such as DNA replication and mitosis (Anand et al. 2023), producing a flatter adaptive landscape in which resistance evolves more gradually but can reach higher levels. These dynamics are consistent with clinical observations: resistance to chemotherapy often emerges more slowly but is frequently more aggressive and associated with resistance to multiple agents (Bukowski et al. 2020). Our results demonstrate that targeted therapies can eliminate tumors with constitutive low evolvability, whose lower ability to generate heritable phenotypic variation prevents evolutionary rescue. In contrast, tumors with constant high evolvability possess a greater capacity to generate resistant phenotypic variants and are therefore more likely to undergo evolutionary rescue under comparable selection pressures. Under these conditions, chemotherapy becomes more effective because the resistance strategies it selects often incur larger fitness costs than those associated with targeted therapy.

We also examined therapeutic responses in populations with plastic evolvability, in which stress‐responsive cellular mechanisms regulate the population's capacity to generate heritable phenotypic variation during therapy (Pienta et al. 2020). This behavior is analogous to stress‐inducible biological systems, where environmentally regulated cellular programs are activated only when beneficial (Fitzgerald et al. 2017; Pribis et al. 2022). Experimentally, such environmentally regulated behavior may arise through stress‐induced mutagenesis, epigenetic remodeling, chromatin reorganization, transcriptional reprogramming, and phenotypic plasticity (Gupta et al. 2019; Fitzgerald et al. 2017). Although these mechanisms operate within individual cells, their collective consequence is an increase in the evolvability of the population under therapeutic stress. By increasing evolvability when advantageous, populations with plastic evolvability effectively balance the benefits of generating higher variation with the costs of evolvability. The plastic‐evolvability population generally attains higher fitness than populations with a fixed evolvability (Enriquez‐Navas et al. 2015).

To target populations with plastic evolvability, we implement an evolutionary double bind, a strategy that exploits the negative covariance between resistance mechanisms, such that resistance to one drug increases sensitivity to the other (Loria et al. 2022; Duarte and Vale 2022; Gatenby et al. 2009). We find that, with the right order and cycle duration of drug administration, the population with plastic evolvability can be driven to extinction. Administering chemotherapy first limits the evolution of resistance while selecting for resistance strategies that carry substantial fitness costs. Following this with the administration of targeted therapy drives the plastic‐evolvability population to extinction. Reversing the treatment order fails to achieve the same outcome, highlighting the importance of treatment sequence in therapy design.

Basanta et al. (2012) had similar findings to ours. Using a matrix game–based model to simulate an evolutionary double bind involving chemotherapy and a *p53* vaccine, they showed that administering chemotherapy first, followed by the vaccine, reduced tumor heterogeneity and improved therapeutic outcomes (Smith 1982; Antonia et al. 2006). More recently, using a Lotka‐Volterra competition model (Bourrat et al. 2024), Luddy et al. (2026) demonstrated that radiation therapy followed by NK cell–based immunotherapy creates a double bind. Radiation selects for cells expressing NK cell ligands, which are effectively targeted by immunotherapy. Experimental validation showed that combination therapy was less effective than sequential therapy. Both studies highlight the critical role of drug order, timing, and duration in designing effective evolutionary therapies.

Our study uses a continuous game‐theoretic framework where resistance is a continuous trait rather than binary (sensitive or resistant) (Vincent and Brown 2005). Our approach is similar in spirit to the work of Cunningham et al. (2011), who incorporated intrinsic and environmental resistance, as well as phenotypic plasticity, to examine the efficacy of double‐bind therapy. We specifically investigate the role of evolvability in shaping therapeutic response and guiding treatment choice. Unlike previous models, ours does not rely on drug sensitization parameters. Instead, we introduce a covariance term that captures how resistance to one drug influences the rate of adaptation to another (Bukkuri and Brown 2021).

Our findings should be interpreted as a proof‐of‐principle demonstrating how differences in tumor evolvability can fundamentally alter optimal therapeutic strategies. While the model predicts that carefully chosen treatment sequences and cycling durations can exploit evolutionary trade‐offs to improve tumor control, translating these schedules into clinical practice presents significant challenges. Successful implementation will require reliable biomarkers of tumor evolvability, longitudinal monitoring of tumor evolutionary dynamics, and treatment protocols capable of adapting to patient‐specific responses (Payne and Wagner 2019; Maley et al. 2017; Passaro et al. 2024). Although several experimental studies have demonstrated the feasibility of sequential evolutionary therapies, further preclinical validation and prospective clinical trials will be necessary before complex adaptive schedules such as those proposed here can be routinely implemented (Luddy et al. 2026; Basanta et al. 2012; Hockings et al. 2025).

Our model has several limitations. First, evolvability is represented phenomenologically through a single population‐level parameter rather than by explicitly modeling the molecular processes that generate heritable phenotypic variation. This abstraction allows us to investigate general evolutionary principles but does not distinguish between the individual contributions of different molecular pathways. Second, we focus on evolutionary adaptation of resistance strategies and do not model therapy‐induced cell‐state transitions or lineage plasticity, which are increasingly recognized as important mechanisms of resistance across multiple cancer types (Subhadarshini et al. 2023; França et al. 2024). Integrating these dynamic cell‐state transitions with eco‐evolutionary models represents an important direction for future work. Third, we isolate the effects of evolvability by holding other ecological parameters constant. In reality, tumors differ in ecological characteristics such as cell turnover, proliferation, and death rates (Gallaher et al. 2019, 2018; Kimmel et al. 2023), which may themselves interact with or be correlated with evolvability and thereby influence therapeutic response. Extending the model to jointly investigate the evolution of resistance together with ecological traits represents another promising avenue for future research. Finally, our model assumes a simplified adaptive landscape and homogeneous tumor population, whereas real tumors exhibit substantial spatial, ecological, and microenvironmental heterogeneity that may further shape both evolvability and therapeutic response (Marzban et al. 2024; Wang et al. 2024). Despite these simplifications, representing evolvability as a population‐level property provides a flexible mathematical framework that can accommodate biological realism as quantitative measurements of tumor evolvability become available.

In summary, there is growing recognition of the role that evolutionary processes play in driving cancer progression and therapeutic resistance. A major focus has been on understanding and quantifying the biological mechanisms that shape a tumor's evolvability and clonal evolution, including genetic mutations, chromosomal instability, epigenetic remodeling, transcriptional reprogramming, and nongenetic phenotypic plasticity (Bakhoum and Cantley 2018; McGranahan and Swanton 2017; Beck et al. 2026). Estimating tumor evolvability will require integrating measurements across multiple levels of biological organization. Fortunately, recent advances in molecular biology and high‐throughput technologies are beginning to make this possible (Payne and Wagner 2019). For example, single‐cell RNA sequencing (scRNA‐seq) and deep mutational scanning allow for high‐resolution mapping of mutational landscapes and evolutionary trajectories within heterogeneous tumors (King et al. 2025; Fowler and Fields 2014). Live‐cell imaging and multiplexed spatial profiling can reveal dynamic phenotypic heterogeneity and cell‐state transitions over time (McGranahan and Swanton 2017; Sengupta and Pratx 2016; Holton et al. 2024; Andreou et al. 2022). Additionally, techniques such as ChIP‐seq and ATAC‐seq provide insights into epigenetic modifications and chromatin accessibility that regulate gene expression variability and potential for adaptation (Yan et al. 2016; Valencia and Kadoch 2019; Corces et al. 2018). These technologies offer an opportunity to estimate tumor evolvability and evaluate its potential as a clinically actionable biomarker.

Plastic evolvability adds an additional layer of complexity to therapeutic response and highlights the limitations of conventional resistance models that assume fixed evolutionary capacities. Our model provides a framework for investigating how such dynamic changes in evolvability influence resistance evolution and therapeutic response. As experimental methods continue to improve our ability to characterize the biological mechanisms underlying tumor evolvability, these measurements can be integrated with eco‐evolutionary models to estimate patient‐specific evolvability, optimize treatment sequencing, and inform evolution‐guided therapeutic design. For strategies such as evolutionary double binds, success depends not only on drug choice but also on the evolutionary trajectories created by treatment order, timing, and duration (Gatenby et al. 2009; Mathur et al. 2022). The present study models evolvability as an environmentally regulated trait; an important future direction will be to treat evolvability itself as an evolvable trait, allowing natural selection to act directly on the biological mechanisms that regulate the generation of heritable phenotypic variation.

As precision oncology continues to advance, incorporating assessments of tumor evolvability into clinical decision‐making may become essential. While more work is needed to translate these insights into practice, evolution‐informed therapy design offers a promising path toward delaying resistance and improving patient outcomes.

## Funding

We appreciate grant support from NIH/NCI 1R01CA258089 and 1U54 CA274507‐01A1, and DoD W81XWH2210680.

## Conflicts of Interest

The authors declare no conflicts of interest.

## Supporting information


**Figure S1:** Comparison of dynamics under chemotherapy: population dynamics (a–c), resistance strategy dynamics (d–f), and evolvability (g–i) dynamics of cancer populations with low, high, and plastic evolvabilities under intermittent therapy. Therapy is administered at *t* = 600 and turned “on” or “off” every 50 time‐steps. Parameters used are shown in Table 1.
**Figure S2:** Comparison of dynamics under combination therapy: population dynamics (a–c), resistance strategy dynamics (d–f), and evolvability (g–l) dynamics of cancer populations with low, high, and plastic evolvabilities under combination therapy. Therapy is initiated at *t* = 600. Dashed lines represent the regimen where the full drug dose is administered; solid lines show the regimen where half of the drug dose is administered to reduce toxicity. Parameters used are shown in Tables 1 and 2.
**Figure S3:** Comparison of dynamics under double‐bind therapy for alternative case: population dynamics (a–c), resistance strategy dynamics (d–f), and evolvability (g–l) dynamics of cancer populations with low, high, and plastic evolvabilities under double‐bind therapy. Therapy is initiated at *t* = 600. Dashed lines represent the regimen where chemotherapy is given first, followed by targeted therapy; solid lines represent the reverse order. The cycle duration is short, that is, therapy is switched every 50 steps. Parameters used are shown in Table S1.
**Figure S4:** Comparison of dynamics under double‐bind therapy for alternative case: population dynamics (a–c), resistance strategy dynamics (d–f), and evolvability (g–l) dynamics of cancer populations with low, high, and plastic evolvabilities under double‐bind therapy. Therapy is initiated at *t* = 600. Solid lines represent the regimen where chemotherapy is given first, followed by targeted therapy; dashed lines represent the reverse order. The cycle duration is long, that is, therapy is switched every 300 steps. Parameters used are shown in Table S1.
**Figure S5:** Comparison of dynamics under combination therapy for alternative case: population dynamics (a–c), resistance strategy dynamics (d–f), and evolvability (g–l) dynamics of cancer populations with low, high, and plastic evolvabilities under combination therapy. Therapy is initiated at *t* = 600. Dashed lines represent the regimen where the full drug dose is administered; solid lines show the regimen where half of the drug dose is administered to reduce toxicity. Parameters used are shown in Table S1.
**Figure S6:** Comparison of dynamics under double‐bind therapy for alternative case: population dynamics (a–c), resistance strategy dynamics (d–f), and evolvability (g–l) dynamics of cancer populations with low, high, and plastic evolvabilities under double‐bind therapy. Therapy is initiated at *t* = 600. Solid lines represent the regimen where chemotherapy is given first, followed by targeted therapy; dashed lines represent the reverse order. In this case, therapy is switched every 500 steps. Parameters used are shown in Table S1.
**Table S1:** Summary of parameters used in the alternative double‐bind simulations. Parameters that have changed from the original model (see Tables 1 and 2) are highlighted in bold.

## Data Availability

No empirical data were used in this study. All results were generated computationally using the mathematical model and simulation code described in the manuscript. All codes used to generate the simulations are available in the GitHub repository: https://github.com/rbhattacharya49/Evolvability‐Determines‐Therapy‐Outcomes.
