# Supplementary material for: Cancer Evolvability Determines Therapy Outcomes
Source: Evol Appl. 2026 Oct 25;19(10):e70320. doi: 10.1111/eva.70320 (PMC13711687; doi:10.1111/eva.70320)
Supplement: Supplementary file 1 [file EVA-19-e70320-s001.docx]

Cancer Evolvability Determines Therapy Outcomes

Supplementary Section

Ranjini Bhattacharya^1,2*,^ Anuraag Bukkuri ^3^, Robert A. Gatenby ^4,5,^, Joel S. Brown^1,5^

1 Department of Integrated Mathematical Oncology, H. Lee Moffitt Cancer Center and Research Institute, Tampa, FL, USA

2 Department of Cancer Biology, University of South Florida, Tampa, FL, USA

3 Department of Mathematics, City St. George’s, University of London, London, UK

4 Department of Radiology, H. Lee Moffitt Cancer Center and Research Institute, Tampa, FL, USA

5 Cancer Biology and Evolution Program, Moffitt Cancer Center and Research Institute, Tampa, FL, USA

Corresponding Author (*) Email: [ranjini.one@gmail.com](mailto:ranjini.one@gmail.com)

Intermittent Therapy

Figure S1: Comparison of dynamics under chemotherapy: Population dynamics (a,b,c), resistance strategy dynamics (d,e,f), and evolvability (g,h,i) dynamics of cancer populations with low, high, and plastic evolvabilities under intermittent therapy. Therapy is administered at t = 600 and turned ‘on’ or ‘off’ every 50 time-steps. Parameters used are shown in Table 1.

Combination Therapy

Figure S2: Comparison of dynamics under combination therapy: Population dynamics (a,b,c), resistance strategy dynamics (d,e,f), and evolvability (g-l) dynamics of cancer populations with low, high, and plastic evolvabilities under combination therapy. Therapy is initiated at $t=600$. Dashed lines represent the regimen where the full drug dose is administered; solid lines show the regimen where half of the drug dose is administered to reduce toxicity. Parameters used are shown in Table 1 & 2.

Alternative Case:

| **Symbol** | **Meaning** | **Value** |
| --- | --- | --- |
| $r$ | Intrinsic growth rate | **0.32** |
| $s_{c}$ | Therapy efficacy of chemotherapy | 0.17 |
| $s_{t}$ | Therapy efficacy of targeted therapy | 0.3 |
| $\sigma_{s_{t}}$ | Targeted therapy efficacy breadth | $\sqrt{2}$ |
| $\sigma_{s_{c}}$ | Chemotherapy efficacy breadth | $\sqrt{6}$ |
| $f_{c}$ | Evolvability scaling factor for chemotherapy | $\left\{ \begin{aligned} \boldsymbol{2}\boldsymbol{.}\boldsymbol{20}\boldsymbol{,}\boldsymbol{if} \mathbf{full} \mathbf{dose} \\ \boldsymbol{4}\boldsymbol{.}\boldsymbol{41}\boldsymbol{,}\boldsymbol{if} \mathbf{half} \mathbf{dose} \end{aligned} \right.$ |
| $f_{t}$ | Evolvability scaling factor for targeted therapy | $\left\{ \begin{aligned} \boldsymbol{1}\boldsymbol{.}\boldsymbol{25}\boldsymbol{,}\boldsymbol{if} \mathbf{full} \mathbf{dose} \\ \boldsymbol{2}\boldsymbol{.}\boldsymbol{5}\boldsymbol{,}\boldsymbol{if} \mathbf{half} \mathbf{dose} \end{aligned} \right.$ |
| $d$ | Cost of evolvability | **0.12** |
| $m$ | Baseline heterogeneity | 0.025 |
| $K_{m}$ | Maximum carrying capacity | 100 |
| $\sigma_{k}$ | Carrying capacity breadth | 10 |
| $\varepsilon$ | Constant evolvability | $\left\{ \begin{aligned} \boldsymbol{0}\boldsymbol{.}\boldsymbol{15}\boldsymbol{,}\boldsymbol{if} \mathbf{low} \mathbf{evolvability} \\ \boldsymbol{0}\boldsymbol{.}\boldsymbol{4}\boldsymbol{,}\boldsymbol{if}\boldsymbol{,}\mathbf{high} \mathbf{evolvability} \end{aligned} \right.$ |
| $\delta$ | Covariance between two resistance strategies | $-$0.015 |

Table S1: Summary of parameters used in the alternative double bind simulations. Parameters that have changed from the original model (see Tables 1 & 2) are highlighted in bold.

Alternative Case: Double Bind

Cycle Duration: 50

Figure S3: Comparison of dynamics under double bind therapy for alternative case: Population dynamics (a,b,c), resistance strategy dynamics (d,e,f), and evolvability (g-l) dynamics of cancer populations with low, high, and plastic evolvabilities under double bind therapy. Therapy is initiated at $t=600$. Dashed lines represent the regimen where chemotherapy is given first, followed by targeted therapy; solid lines represent the reverse order. The cycle duration is **short**, i.e., therapy is switched every **50** steps. Parameters used are shown in Table S1.

Alternative Case: Double Bind

Cycle Duration: 300

Figure S4: Comparison of dynamics under double bind therapy for alternative case: Population dynamics (a,b,c), resistance strategy dynamics (d,e,f), and evolvability (g-l) dynamics of cancer populations with low, high, and plastic evolvabilities under double bind therapy. Therapy is initiated at $t=600$. Solid lines represent the regimen where chemotherapy is given first, followed by targeted therapy; dashed lines represent the reverse order. The cycle duration is **long**, i.e., therapy is switched every **300** steps. Parameters used are shown in Table S1.

Alternative Case: Combination Therapy

Figure S5: Comparison of dynamics under combination therapy for alternative case: Population dynamics (a,b,c), resistance strategy dynamics (d,e,f), and evolvability (g-l) dynamics of cancer populations with low, high, and plastic evolvabilities under combination therapy. Therapy is initiated at $t=600$. Dashed lines represent the regimen where the full drug dose is administered; solid lines show the regimen where half of the drug dose is administered to reduce toxicity. Parameters used are shown in Table S1.

Alternative Case: Double Bind

Cycle Duration: 500

Figure S6: Comparison of dynamics under double bind therapy for alternative case: Population dynamics (a,b,c), resistance strategy dynamics (d,e,f), and evolvability (g-l) dynamics of cancer populations with low, high, and plastic evolvabilities under double bind therapy. Therapy is initiated at $t=600$. Solid lines represent the regimen where chemotherapy is given first, followed by targeted therapy; dashed lines represent the reverse order. In this case therapy is switched every **500** steps. Parameters used are shown in Table S1.
